# Effectiveness of preoperative staging in rectal cancer: digital rectal examination, endoluminal ultrasound or magnetic resonance imaging?

**DOI:** 10.1038/sj.bjc.6601871

**Published:** 2004-06-08

**Authors:** G Brown, S Davies, G T Williams, M W Bourne, R G Newcombe, A G Radcliffe, J Blethyn, N S Dallimore, B I Rees, C J Phillips, T S Maughan

**Affiliations:** 1 Department of Radiology, University Hospital of Wales, Cardiff, UK; 2 Centre for Health Economics and Policy Studies, School of Health Science, University of Wales, Swansea, UK; 3 Department of Pathology, University of Wales College of Medicine, Cardiff, UK; 4 Department of Medical Computing and Statistics, University of Wales College of Medicine, Cardiff, UK; 5 Departments of Surgery, Llandough Hospital, Penarth, UK; 6 Department of Radiology, Llandough Hospital, Penarth, UK; 7 Department of Histopathology, Llandough Hospital, Penarth, UK; 8 Department of Surgery, University Hospital of Wales, Cardiff, UK; 9 Department of Clinical Oncology, Velindre Hospital, Cardiff, UK

**Keywords:** rectal neoplasm staging, endosonography, magnetic resonance imaging, comparative study, cost–benefit analysis, rectal neoplasms therapy, radiotherapy, surgery, pathology

## Abstract

In rectal cancer, preoperative staging should identify early tumours suitable for treatment by surgery alone and locally advanced tumours that require therapy to induce tumour regression from the potential resection margin. Currently, local staging can be performed by digital rectal examination (DRE), endoluminal ultrasound (EUS) or magnetic resonance imaging (MRI). Each staging method was compared for clinical benefit and cost-effectiveness. The accuracy of high-resolution MRI, DRE and EUS in identifying favourable, unfavourable and locally advanced rectal carcinomas in 98 patients undergoing total mesorectal excision was compared prospectively against the resection specimen pathological as the gold standard. Agreement between each staging modality with pathology assessment of tumour favourability was calculated with the chance-corrected agreement given as the kappa statistic, based on marginal homogenised data. Differences in effectiveness of the staging modalities were compared with differences in costs of the staging modalities to generate cost effectiveness ratios. Agreement between staging and histologic assessment of tumour favourability was 94% for MRI (*κ*=0.81, s.e.=0.05; *κ*_*W*_=0.83), compared with very poor agreements of 65% for DRE (*κ*=0.08, s.e.=0.068, *κ*_*W*_=0.16) and 69% for EUS (*κ*=0.17, s.e.=0.065, *κ*_*W*_=0.17). The resource benefits resulting from the use of MRI rather than DRE was £67164 and £92244 when MRI was used rather than EUS. Magnetic resonance imaging dominated both DRE and EUS on cost and clinical effectiveness by selecting appropriate patients for neoadjuvant therapy and justifies its use for local staging of rectal cancer patients.

Meticulous surgical technique, as exemplified by total mesorectal excision (TME), has improved survival by reducing local recurrences in patients undergoing resection with curative intent (Heald, 1995; Carlsen *et al*, 1998). In addition, preoperative neoadjuvant radiotherapy and chemoradiotherapy regimens also promise to improve survival (Marsh *et al*, 1994; Anonymous, 1997; Pahlman *et al*, 1998; Dahlberg *et al*, 1999), but their success needs to be tempered against the inevitable morbidity associated with such treatments (Dahlberg *et al*, 1998). In 1997, the Scandinavian rectal cancer trials (Anonymous, 1997) showed that preoperative therapy reduced local recurrence rates in patients undergoing rectal cancer surgery. This effect was also shown in patients undergoing TME with significantly reduced local recurrence rates in patients undergoing TME surgery plus preoperative radiotherapy compared with patients undergoing TME surgery alone (Kapiteijn *et al*, 2001). However, preoperative short-course radiotherapy had no effect on local recurrence rates in patients with positive circumferential resection margin (CRM) status. Both trials suggest that preoperative radiotherapy can reduce local recurrence rates compared with surgery alone, but it is clear that the magnitude of the benefit is very small if such treatments are used unselectively (van de Velde, 2002).

In order to improve patient selection, a preoperative staging method is required to identify early stage tumours that are suitable for treatment by surgery alone as well as more advanced tumours that require more intensive therapy than short-course radiotherapy to enable tumour regression from the potential surgical resection margins. Clinical assessment by digital rectal examination (DRE) and endoluminal ultrasound (EUS) are recommended as a method of making this preoperative assessment (1996).

It has previously been shown that using a high-resolution technique, thin slice magnetic resonance imaging (MRI) can be used to measure the depth of extramural spread accurately with good correlation with corresponding pathology measurements in resection specimens (Brown *et al*, 1999). Furthermore, the relationship of tumour to the mesorectal fascia can be seen so that CRM positive status is predicted when tumour is imaged within 1 mm of the mesorectal fascia (Brown *et al*, 2003a). However, MRI is generally regarded as an expensive modality that has not proven clinically effective or cost-effective in oncology imaging. Given that DRE and EUS are widely available and are the current standards for preoperative assessment of local tumour stage in many centres, a prospective comparison of the clinical value of MRI in staging rectal cancer patients over these techniques was performed.

In this study, the accuracy of high-resolution MRI, DRE and EUS in identifying favourable, unfavourable and locally advanced rectal carcinomas was compared prospectively against the gold standard of the pathological findings in the resection specimens. The potential impact of each staging modality on the preoperative treatment pathway was then compared, for clinical benefit and cost-effectiveness.

## MATERIALS AND METHODS

### Patients

Patients with biopsy diagnosed rectal cancer referred our institution were eligible for informed consent to participate in this study. This study was performed according to a protocol approved by the Ethics Committee of our institution and informed consent was obtained from each patient.

Digital rectal examination was performed as an outpatient clinical assessment by the consultant colorectal surgeon (AGR, BIR) as the initial assessment of the primary tumour. Colonoscopy examination findings and examination under general anaesthesia were not included in the analysis of clinical effectiveness nor in the costings.

The fixity of the tumour was recorded as mobile, tethered or fixed according to criteria of Nicholls *et al* (1985). EUS and MRI were undertaken within 2 weeks of TME surgery. A total of 26 women and 72 men (age range 28–89 years) consented to enter the study over a 3-year period.

### Imaging technique

Both EUS and MRI examinations were performed with knowledge of the height of the tumour from the anal verge but without knowledge of any other investigation findings.

EUS was performed by a single observer (JB, a consultant radiologist experienced in EUS) using a 7.5/10 MHz radial scanning transducer with water filled probe cover. EUS staging was performed according to established criteria (Beynon *et al*, 1986) using the T and N components of the TNM classification. For T3 and T4 tumours, the maximum depth of spread in mm beyond the muscularis propria was recorded.

T2-weighted MRI was performed (Brown *et al*, 1999). The tumour was staged according to the TNM classification and the maximum depth of extramural spread was recorded using criteria developed previously (Brown *et al*, 1999, 2003b). In addition, note was made of the relationship of tumour (either in continuity with the primary mass or as a separate mesorectal deposit (Brown *et al*, 2003b) with an irregular border or a mixed intensity signal) to the mesorectal fascia. Cases with tumour reaching within 1 mm of this landmark, or beyond it, were defined as CRM positive.

In assessing extramural spread, cases were classified into favourable stage, unfavourable or locally advanced stage (depending on T stage and nodal status) and advanced stage (if the potential CRM was at risk). Table 1

**Table 1:** Preoperative stage groupings using DRE, EUS and MRI

	**Favourable**	**No favourable features identified**	**Locally advanced**
DRE	Mobile	(1) Mobile <5 cm from anal verge	Fixed
		(2) Tethered tumour	
		(3) Not assessable due to pain or beyond DRE and sigmoidoscopic assessment of mobility	
			
EUS	T1N0, T2N0 or 0T3<1 mm N0	Node positive	T4
		T3>1 mm	
		Tumour not assessable due to bulk or location beyond the edge of the probe	
			
MRI	T1N0, T2N0 or T3<1 mm N0	Node positive	T4 or tumour ⩽1 mm from mesorectal fascia
		T3>1 mm	
			
Histopathology	pT1N0, pT2N0 or pT3<1 mm N0	Node positive	pT4 or tumour ⩽1 mm from the CRM
		T3>1 mm	

shows the criteria for allocating patients into these three local stage categories and the therapeutic plan resulting from this evaluation.

### Treatment schedules

TME surgery was performed in all cases, with sphincter sparing anterior resection in 75 patients and abdominoperineal excision in 23 patients. Patients judged to have favourable tumours were treated with surgery only. Those with unfavourable tumours received a 1-week course of preoperative radiotherapy of 25 Gy in five fractions in 5 days using a three field technique on 10 Mv linear accelerators in the week immediately preceding surgery (Anonymous, 1997). Patients with locally advanced, clinically inoperable tumours were given a long course of radiotherapy using a three field planned treatment to the posterior pelvis giving 45 Gy in 25 fractions over 5 weeks in combination with infusional 5FU (200 mg m^−2^ throughout radiotherapy) followed by surgery. DRE, EUS and MRI were performed at baseline and repeated within 2 weeks prior to surgery to provide data for correlation with the resection specimen.

### Pathology

Pathological staging was undertaken by a consultant pathologist according to the TNM classification and the pathological extent of maximal extramural spread in mm and the distance of tumour to the nearest CRM was measured.

### Cost analysis

The costs of staging for rectal cancer depend on which perspective is employed and include staff time, equipment costs, running costs, overheads, patients costs, etc. In addition, there are the implications of successful and accurate staging and more pertinently the cost implications resulting from unsuccessful and inaccurate staging. The implications of unsuccessful and inaccurate staging on resource utilisation and patients’ quality of life are extensive. For this analysis, calculations were restricted to the impact on preoperative radiotherapy budgets.

The treatment strategy model employed recommendations from the Swedish rectal cancer (Anonymous, 1997) trial and the Dutch TME trial (Kapiteijn *et al*, 2001), which are summarised in Table 1. Thus, all operable patients are eligible to receive short-course high-dose preoperative radiotherapy (25 Gy in five fractions over 5 days) but favourable tumours defined as node negative: T1, T2 or T3a cases that can undergo curative resection by optimised surgery alone. In addition, patients considered to have locally advanced disease receive long-course radiotherapy (45 Gy in 25 fractions over 5 weeks).

Estimates of the local procedure costs for EUS and MRI were adjusted to account for variation in other centres in the sensitivity analysis. The costs of short- and long-course radiotherapy and chemotherapy sessions were obtained from the participating oncology centre, Velindre Hospital (Table 2

**Table 2:** Procedure and radiotherapy costs

**Procedure costs**	**£**
DRE	0
EUS	78
MRI	130
*Adjuvant therapy*	
Radiotherapy session	84
Chemotherapy session	180
Short-course radiotherapy treatment (5 sessions)	420
Long-course radiotherapy treatment (25 sessions)	2100

). The costs of overstaging (£2538) were based on the cost of preoperative adjuvant treatment provided unnecessarily, while the costs of understaging were based on estimates of treatment for recurrences in the literature (Neymark and Adriaenssen, 1999) (assumed to be between £8460 and £14 840) multiplied by the probability of recurrence (30%) (Frykholm *et al*, 1995). The differences in rates of effectiveness for the staging modalities were used to generate comparisons of the resource implications, in terms of radiotherapy costs for the 98 patients studied.

### Cost effectiveness

Differences in rates of effectiveness of the staging modalities were compared with differences in the costs of the staging modalities to generate cost effectiveness ratios and a series of one-way sensitivity analyses were carried out on the baseline findings.

### Statistical methods

Agreement between EUS, MRI or DRE with pathology assessment of tumour favourability was calculated with the chance-corrected agreement given as the kappa statistic, based on marginal homogenised data (Scott, 1955).

## RESULTS

The study cohort represented a consecutive series of patients referred with rectal cancer consenting to take part in the study from two surgical centres in one city. In total, 23 patients had tumour <6 cm to the anal verge, 12 had tumour wholly above the peritoneal reflection and the remainder were located in the mid-rectum. Assessment of tumour mobility was achieved in 74 patients, the remaining 24 having tumours that were too high or too painful to assess by DRE. Only 54 were adequately evaluated by EUS: five patients were unassessable due to failed bowel preparation, the full length of the tumour was beyond the EUS probe in 28 and the remaining 11 patients experienced severe pain or declined the procedure. All 98 patients tolerated the MRI examination.

In total, 48 operable patients underwent resection within 2 weeks of clinical and radiological assessment. A further 44 patients underwent short-course high-dose preoperative radiotherapy and six patients with clinically inoperable tumours received long-course preoperative chemoradiotherapy.

### Clinical effectiveness

Pathological examination of resected specimens showed 31 patients to have favourable tumours (T1, T2, T3<1 mm spread beyond the outer longitudinal muscle coat and N0 disease), 39 patients having unfavourable (T3>1 mm or node positive), and the remaining 28 patients having locally advanced tumours (pT4 or CRM positive). Table 3

**Table 3:** DRE, EUS and MRI assessment of ‘correct’ preoperative treatment strategy *vs* histopathology gold standard

	**Histopathology**			
	**Favourable**	**Unfavourable**	**Locally advanced**	**Total**
*DRE assessment*				
Surgery alone	**22**	22	7	51
Short-course RT	7	**14**	18	39
Long course RT	2	3	**3**	8
Total	31	39	28	98
				
*EUS assessment*				
Surgery alone	**14**	5	8	27
Short-course RT	17	**32**	19	68
Long course RT	0	2	**1**	3
Total	31	39	28	98
				
*MRI assessment*				
Surgery alone	**31**	5	2	38
Short-course RT	0	**33**	4	37
Long-course RT	0	1	**22**	23
Total	31	39	28	98

Values in bold indicate number of cases showing agreement with histopathology.

shows the agreement between each of the staging modalities with the pathology gold standard.

### Preoperative identification of favourable prognosis tumours

Digital rectal examination correctly identified 22 out of 31 (71%) patients with favourable prognosis tumours. Four favourable prognosis mobile tumours were not identified because tumour sited high in the rectum was beyond the reach of DRE. In a further three cases apparent tethering on clinical examination indicating more extensive extramural spread was not confirmed on histological examination. In two cases low bulky tumours that were deemed fixed on clinical assessment were confined to the rectal wall on subsequent histopathological assessment. Review of both cases on MR imaging showed bulky polypoidal tumours adjacent to a markedly enlarged prostate gland.

Endoluminal ultrasound correctly identified 14 of 31 (45%) favourable prognosis tumours. In two patients, extramural depth had been substantially overestimated. In both cases, large intraluminal tumour masses had been located in the upper rectum. Review of the study showed that the size and position of the tumour had resulted in tangential placement of the probe against the tumour. The resultant obliquity of the probe had produced artefactual loss of the outer muscle coat resulting in overestimation of tumour depth. In the remaining 15 patients, failure to reach tumour using the EUS probe resulted in inability to assess tumour depth.

**Figure 1:**
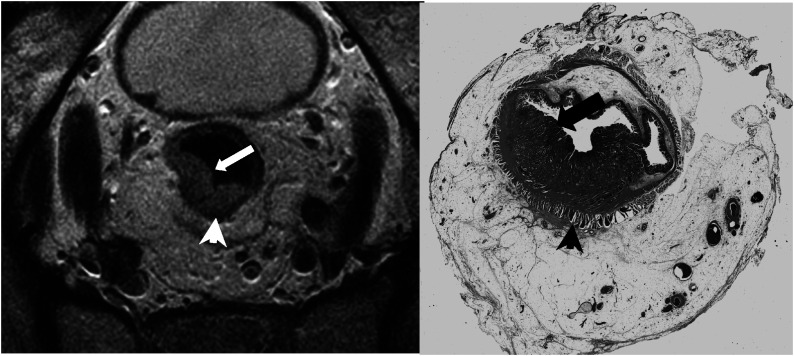
Favourable tumour. High-resolution T2-weighted fast spin-echo image and corresponding histological (H&E stained) wholemount section. The tumour (arrow) is depicted as a U-shaped polypoidal mass of intermediate signal intensity. The muscualris propria is of lower signal intensity (arrow head) and does not appear breached by tumour indicating tumour confined to bowel wall (T2). The corresponding wholemount histology section confirms that this is a T2 tumour.

Magnetic resonance imaging correctly identified all patients with favourable prognosis tumours (example shown in Figure 1

); however, it was noted that in nine out of 31 patients there was overlap between MR and histology assessment of T2 *vs* T3<1 mm spread.

### Preoperative identification of unfavourable prognosis tumours

Histopathological examination showed tumour extension into perirectal fat and/or node-positive status in 39 patients. Clinical assessment correctly identified 14 out of 39 (36%). In 22 out of 39 patients clinical assessment judged tumours as mobile and nine out of 22 showed tumour spread >5 mm into perirectal fat that was not clinically tethered. In three out of 39 patients, clinical staging suggested tumour fixation. Corresponding MR imaging in these three patients showed extramural low signal intensity indicating fibrosis and subsequent histopathological assessment confirmed extensive peritumoral fibrosis and inflammatory change as a cause of apparent fixation on DRE.

EUS assessment resulted in the correct identification of 32 out of 39 (82%) patients with unfavourable prognosis tumours. Two patients that had been endoscopically overstaged were shown on EUS as showing invasion of adjacent bladder and prostate. In both cases hyporeflective tumour produced loss of definition of the borders between rectum and adjacent organ. These changes were shown to relate to extramural fibrosis but not tumour. In three cases staged as favourable tumours by EUS, nodal metastases were not detected by EUS. On review of the pathology specimens, the nodal metastases were sited >30 mm from the bowel wall and thus not encompassed within the EUS field of view. In two further cases, both low bulky tumours, extramural tumour depth of >1 mm had been underestimated.

**Figure 2:**
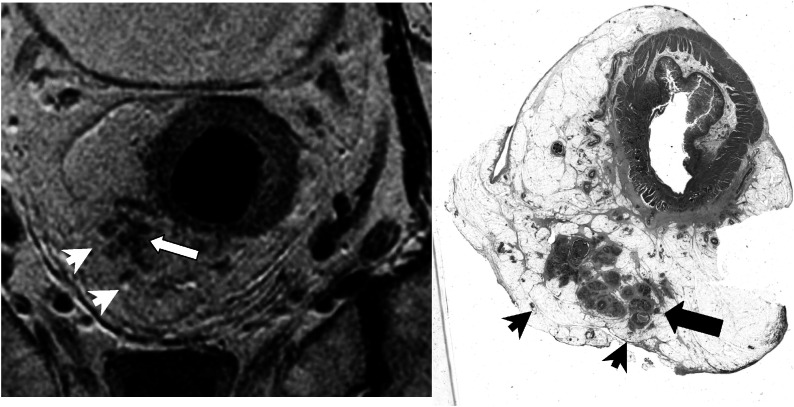
Unfavourable prognosis tumour. High-resolution T2-weighted fast spin-echo image and corresponding histological (H&E stained) wholemount section. The MRI scan shows widespread discontinuous tumour deposits (arrows) (representing either nodes replaced by tumour or tumour satellites) within the mesorectum, but not extending to the mesorectal fascia (arrow heads). This is confirmed as node-positive disease on corresponding wholemount histology section.

Using MRI, 33 out of 39 (85%) unfavourable prognosis tumours were correctly identified (example shown in Figure 2

). In one patient, positive nodes were shown on MRI to lie within 1 mm of the mesorectal fascia and therefore classified as advanced (potential CRM positive), but subsequent histological examination showed that this distance was 2 mm (CRM negative) indicating this was unfavourable tumour. In two cases, node-positive status indicating unfavourable prognosis tumour was not detected by MRI. In each of these cases, the tumour was within 5 cm of the anal verge and imaging had not encompassed the upper third of the mesorectum containing these positive nodes. In the remaining three out of 39 patients, tumour spread >1 mm had been underestimated by MRI.

### Preoperative identification of locally advanced tumours

There were 28 cases with pathological features indicating locally advanced disease. Only three of these were successfully identified by clinical staging. Of these 28 cases, 18 were classified as unfavourable: five were too high for clinical assessment, six tumours were mobile and less than 5 cm from the anal verge and seven tumours tethered on examination, but on subsequent histopathology examination showed extensive extramural spread or nodes involving the CRM. A further seven were classified as favourable as they were clinically mobile and >5 cm from the anal verge.

Only one locally advanced case was successfully identified using EUS. In this case, low tumour with invasion into the levator was demonstrated. In 12 patients, tumour was unassessable because tumour could not be reached by the probe or because of pain experienced by the patient. In 15 patients, tumour deposits involving the mesorectal fascia resulting in positive CRM had not been identified.

**Figure 3:**
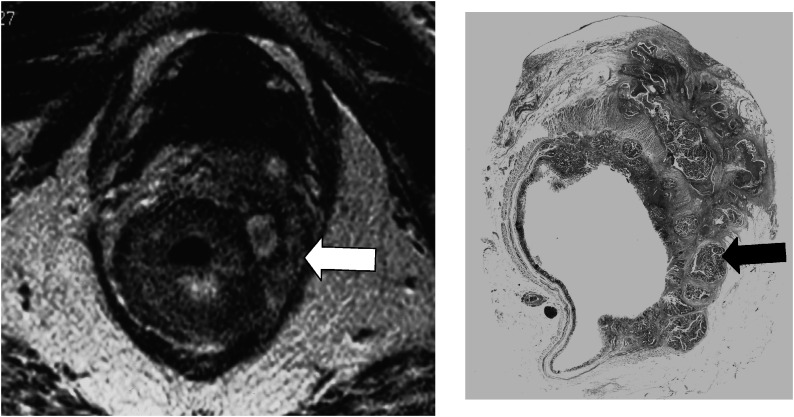
Locally advanced tumour. High-resolution T2-weighted fast spin-echo image and corresponding histological (H&E stained) wholemount section. This shows tumour extending beyond the bowel wall and involving the potential left lateral resection margin (arrow). Margin involvement is confirmed on subsequent histopathological section (arrow).

Of 28 patients, 22 with locally advanced tumours were successfully identified using MRI (example shown in Figure 3

). In four patients, nodes close to the mesorectal fascia had not been detected. In each of these cases the nodes were partially replaced by small tumour foci that were not resolved on MR images. In two patients tumour was thought to have breached the wall anteriorly by <1 mm, but histopathologic examination showed stage pT4 peritoneal infiltration by tumour.

There was a high level of agreement between MRI and histologic assessment of tumour favourability (94%) (*κ*=0.81, standard error=0.05; weighted *κ*=0.83). This compared with very poor agreement between DRE and histologic assessment of tumour favourability (65%) (*κ*=0.08, standard error=0.068, weighted *κ*=0.16), and a poor level of agreement between EUS and histologic assessment of tumour favourability (69%) (*κ*=0.17, standard error=0.065, weighted *κ* 0.17).

### Treatment

Table 4

**Table 4:** costs incurred as a result of incorrect preoperative treatment on the basis of either DRE, EUS and MRI assessment

	**DRE**		**EUS**		**MRI**	
**Treatment cost**	**Number of patients**	**Total cost £**	**Number of patients**	**Total cost £**	**Number of patients**	**Total cost £**
Long-course RT instead of short-course RT (£1680 per incorrectly staged patient)	3	5040	2	3360	1	1680
Short-course instead of long-course RT (£2538 per incorrectly staged patient)	18	45 684	19	48 222	4	10 152
						
Long-course RT instead of surgery alone (£2100 per incorrectly staged patient)	2	4200	0	0	0	0
Surgery alone instead of long-course RT (£2538 per incorrectly staged patient)	7	17 766	8	20 304	2	5076
Surgery alone instead of short course RT (£420 per incorrectly staged patient)	22	9240	5	2100	5	2100
Short-course RT instead of surgery alone (£2538 per incorrectly staged patient)	7	17 766	17	43 146	0	0
						
Total incorrectly staged	59	99 696	51	117 132	12	19 008

indicates the impact these errors in staging would have in clinical practice given the three different treatment schedules in the model. On DRE staging alone, 51 patients would have had surgery alone, 39 short-course RT and eight long-course RT. In comparison with the gold standard, 22, 14 and three patients in each treatment group respectively were appropriately selected (40%). The remainder would have been either under or over treated.

On EUS staging, 47 patients (48%) would have been correctly selected, while using MRI 86 patients (88%) would have had the appropriate treatment selected.

The costs incurred as a result of incorrect preoperative treatment on the basis of either DRE, EUS and MRI assessment are shown in Table 4. The resource benefits that accompany each additional successful and accurately staged patient amount to £1282 if MRI is used instead of DRE and £1195 if MRI is used instead of EUS, indicating that MRI is a cost-effective technique.

The total costs incurred (procedure costs and costs resulting from incorrect preoperative treatment) from staging the 98 patients using the three modalities are shown in Table 5

**Table 5:** Total costs of staging using three modalities

	**DRE**	**EUS**	**MRI**
Procedure costs	0	7644	13 524
Costs of incorrect staging	99 696	117 132	19 008
Total costs	99 696	124 776	32 532
			
*Total cost per staged patient*	*1017*	*1273*	*332*

. The resource benefits that result from the use of MRI rather than DRE amount to £67164 and £92244 when MRI is used rather than EUS. In addition, MRI correctly staged 86 patients, 47 more than DRE and 39 more than EUS. In terms of cost-effectiveness MRI dominates both DRE and EUS on the grounds of cost and effectiveness.

### Sensitivity analysis

#### Assuming the resource implications of understaging are zero

This scenario assumes that there are no resource implications resulting from understaging. The total cost of staging using MRI amounts to £15204 compared with £54150 using EUS and £27006 for DRE. Therefore, MRI dominates both DRE and EUS in terms of cost and effectiveness.

#### Increasing the cost of an MRI to £500

Given the variation in the alleged cost of an MRI, this scenario assumes that the cost of an MRI is £500. The cost of staging 98 patients using MRI (including the resource implications from incorrect staging) then amounts to £68008 – a resource difference of £56768 compared with EUS and £31688 compared with DRE. Thus, in order for the total cost for MRI to equate with EUS (taking into account the resource implications of incorrect staging), the cost of an MRI procedure would need to be substantially higher, that is, £1079.

#### Only including procedure costs

If only procedure costs were included and the resource implications resulting from incorrect staging ignored, the total cost for EUS would be £7644 and for MRI the cost would be £13 524, a difference of £5880. For this additional expenditure there are 39 more patients who are successfully and accurately staged by using MRI than EUS and 47 when compared with DRE. This produces a cost per additional successful and accurately staged patient of £151 when using MRI rather than EUS and £288 when compared with DRE. When the procedure cost of MRI is £500 the cost per additional correctly staged patient is £1060 (MRI vs EUS) and £1043 (MRI *vs* DRE), which appears to represent a good investment, given that the cost of treating recurrence is £8460 and we have assumed that the probability of recurrence for understaging is 30%.

## DISCUSSION

This study, employing meticulous histopathological assessment of tumour staging as the gold standard, clearly demonstrates the benefits of thin slice, T2 weighted, high-resolution MRI over both DRE and EUS for staging rectal cancer preoperatively. The study highlights the value of employing a noninvasive technique as inability to assess high rectal tumours hampered the accuracy of both EUS and DRE in identifying favourable, unfavourable and advanced tumours of the upper rectum. MRI was able to accurately identify all favourable prognosis tumours regardless of height from the anal verge. This improved accuracy of assessment translates into better patient selection by diminishing the use of needless and potentially harmful neoadjuvant therapy in patients with node negative status and <1 mm extramural tumour spread who have a favourable prognosis (Willett *et al*, 1999). Moreover, the MRI images allow visualisation of the whole of the tumour, its anatomical disposition in any plane extramurally, and its relationship to the CRM, all of which greatly assist the planning of any preoperative radiotherapy and the surgical resection itself.

While there is growing evidence that preoperative radiotherapy and TME have an additive (Holm *et al*, 1997) effect on improvement of local recurrence rates, it is becoming clear that preoperative radiotherapy for ‘good’ prognosis patients is overtreatment that wastes resources and leads to significant morbidity. Many studies have shown that the depth of extramural invasion, nodal involvement and CRM involvement are independent markers of poor prognosis (Cawthorn *et al*, 1986; Jass and Love, 1989; Adam *et al*, 1994; Hall *et al*, 1998) and selection for neoadjuvant therapy is being increasingly based on these. MRI performs particularly well over other modalities in the assessment of these parameters and studies have prospectively validated the technique for accuracy of depth of extramural spread and its ability to predict CRM involvement (Brown *et al*, 1999, 2003a). By contrast, DRE (which depends on the subjective appreciation of tumour mobility or fixity) performs poorly, understaging 47% of cases. EUS tends to overestimate tumour depth (Akasu *et al*, 1996) due to the obliquity of the probe in relation to the lesion and difficulty in separating peritumoural inflammation or fibrosis from true tumour (Maier *et al*, 1997). No previous EUS studies have assessed its accuracy in TME specimens yet, in the present study, its inherent small field of view limited its usefulness in assessing the whole mesorectum. Neither nodes/tumour deposits located within the mesorectum at a distance from the bowel lumen nor the mesorectal fascia (representing the potential CRM) were visualised. Thus, neither EUS nor DRE were able to assess discontinuous mesorectal tumour deposits that might govern operability for cure, and both are invasive, potentially painful modalities that cannot be applied to all patients for technical reasons.

Relatively few early lesions occurred in this study (representing our typical experience of symptomatic rectal cancer). This may account for the relatively poor performance of EUS at T staging. The assessment of early tumours by EUS was outside the scope of this study.

Nevertheless, MRI appeared to be poor at separating T1 from T2 tumours and in distinguishing sessile or polypoid adenomas from T1 adenocarcinomas, whereas others have shown that EUS has been is highly accurate in the assessment of early tumours (Akasu *et al*, 2000). Clearly, EUS has an important and complementary role in staging early lesions. Our data therefore suggest that MRI is a poor technique for selecting patients for local excision, but future studies should address the role of EUS in this subgroup of patients.

MRI shows clear clinical benefits over the traditional method using combined DRE and EUS in terms of correct allocation of patients to treatment groups for radiotherapy and/or chemotherapy. Accordingly, MRI results in significant treatment cost benefits that are very likely to offset the costs of the procedure itself as well as being more clinically effective than the alternatives.

Thus, the advantages of preoperative high-resolution MRI in selecting appropriate patients for neoadjuvant therapy justify its routine use in the local staging of rectal cancer patients.
